# Study of lipase producing gene in wheat – an in silico approach

**DOI:** 10.1186/s43141-021-00150-1

**Published:** 2021-05-17

**Authors:** Shradha Rani, Priya Kumari, Raju Poddar, Soham Chattopadhyay

**Affiliations:** Department of Bio-Engineering, Birla Institute of Technology, Mesra, Ranchi, 835215 India

**Keywords:** Lipase, Plants database, Homology modeling, Molecular dynamic simulations, Docking, Binding efficiency

## Abstract

**Background:**

Lipases (EC 3.1.1.3) catalyze the hydrolysis of oil into free fatty acids and glycerol forming the 3rd largest group of commercialized enzymes. Plant lipases grab attention recently because of their specificity, less production and purified cost, and easy availability. In silico approach is the first step to identify different genes coding for lipase in a most common indigenous plant, wheat, to explore the possibility of this plant as an alternative source for commercial lipase production. As the hierarchy organization of genes reflects an ancient process of gene duplication and divergence, many of the theoretical and analytical tools of the phylogenetic systematics can be utilized for comparative genomic studies. Also, in addition to experimental identification and characterization of genes, for computational genomic analysis, Arabidopsis has become a popular strategy to identify crop genes which are economically important, as Arabidopsis genes had been well identified and characterized for lipase. A number of articles had been reported in which genes of wheat have shown strong homology with Arabidopsis. The complete genome sequences of rice and Arabidopsis constitute a valuable resource for comparative genome analysis as they are representatives of the two major evolutionary lineages within the angiosperms. Here, in this in silico approach, Arabidopsis and *Oryza sativa* serve as models for dicotyledonous and monocotyledonous species, respectively, and the genomic sequence data available was used to identify the lipase genes in wheat.

**Results:**

In this present study, Ensembl Plants database was explored for lipase producing gene present in wheat genome and 21 genes were screened down as they contain specific domain and motif for lipase (GXSXG). According to the evolutionary analysis, it was found that the gene TraesCS5B02G157100, located in 5B chromosome, has 58.35% sequence similarity with the reported lipase gene of *Arabidopsis thaliana* and gene TraesCS3A02G463500 located in the 3A chromosome has 51.74% sequence similarity with the reported lipase gene of *Oryza sativa*. Homology modeling was performed using protein sequences coded by aforementioned genes and optimized by molecular dynamic simulations. Further with the help of molecular docking of modeled structures with tributyrin, binding efficiency was checked, and the difference in energies (DE) was −9.83 kcal/mol and −6.67 kcal/mol, respectively.

**Conclusions:**

The present work provides a basic understanding of the gene-encoding lipase in wheat, which could be easily accessible and used as a potent industrial enzyme. The study enlightens another direction which can be used further to explore plant lipases.

## Background

**Fig. 1:**
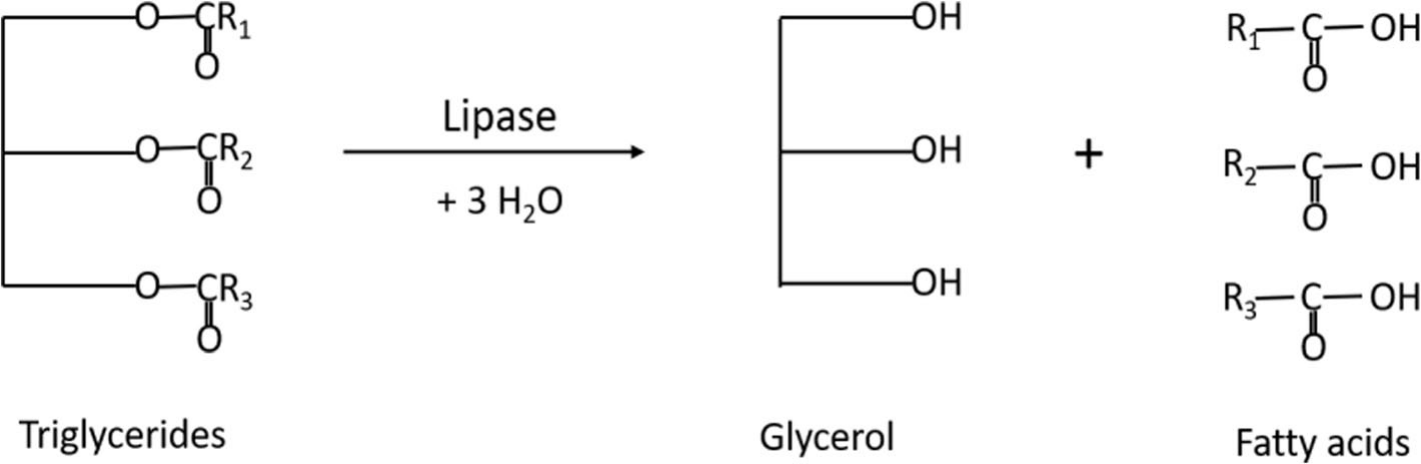
Hydrolysis of triglycerides in the presence of lipases

Lipases are ubiquitous enzymes, widespread in nature, and it can be of microbial (bacterial, fungal, and yeast), animal, or plant origin. Lipase (EC 3.1.1.3), a member of the lipolytic enzyme family, catalyzes the hydrolysis of the ester bonds of tri-, di-, and monoglycerides into fatty acids and glycerol [6] as described in Fig. 1.

In the active site of the enzyme lipase, the catalytic triad serine, aspartate or glutamate and histidine, is present, in which serine acts as a nucleophile and aspartate or glutamate acts as a catalytic acid residue and forms a hydrogen bond with histidine. Lipases consist of a pentapeptide consensus motif (Gly-X-Ser-X-Gly) [12, 17].

Recently, plant lipases have been the focus of much attention as biocatalysts. It presents advantages over microbial lipases due to specificity, low production cost, availability, and ease of purification [37]. Plant lipases are often present in the reserve tissues of germinating seedlings or in tissues with a large amount of triacylglycerols, where they play an important role in biological reactions such as lipolysis, esterification, and transesterification and thus helping in plant growth and development. In higher plants, triacylglycerols (TAGs) may be in few percentages of total lipids in the leaf tissue but can make up to 60% of the dry weight of oil seeds. Fatty acids can be cleaved off by a lipase and further metabolized in peroxisomes through b-oxidation to yield acetyl-coA [20, 31]. A minor application of the lipase enzyme is that it is used as a diagnostic tool in medicine. Apart from this, it is used in the food, detergent, and pharmaceutical sectors. The variety of lipase applications led to increased research to characterize them and better understand their kinetics and reaction mechanisms and to establish methods for lipase production in homologous and heterologous expression systems. In the worldwide enzyme industry market, the rank of lipases has grown significantly high. It is also believed that in the near future, it will acquire importance as comparable to that of the peptidases, which represent 25 to 40% of industrial enzyme sales [13, 16]. A number of articles have been published, especially concerning the synthesis of seed lipases from barley, linseed, maize, rice, and wheat [3, 4, 28, 30]. Researchers have also studied for optimization of different physicochemical conditions and estimated the lipase activity and purified them.

In India, bread wheat (*Triticum aestivum* L.) is one of the most widely grown wheat species, occupying 37% of the total cultivated land. Many vitamins, essential amino acids, and proteins are present in wheat germs [14]. Although a number of articles have been reported on the purification and characterization of lipase from wheat, very few reports are available on the lipase gene present in wheat and its biological reaction with substrates [19, 36].

To understand plant lipase in more detail, the major focus of our study is to identify lipase genes from the wheat genome which can be used as a basis for further applied researches. In this present study, we have identified and located genes present in the wheat genome, through in silico study. These identified protein sequences were further modeled and docked to check their biological activity. Further, the discovery of putative lipase gene sequences presents in the wheat using bioinformatics tools has been described.

## Methods

### Sequence retrieval and analysis

Data available at Ensembl Plants database [5] was mined by using “lipase” as a keyword to search for all the lipase genes present in the annotated *Triticum aestivum* genome (access date November 10, 2019).

### Motif and domain search

By using the Scan Prosite tool [8] found at ExPASy-PROSITE, the motif of lipase genes was analyzed and sequences with the GXSXG lipase motif [38] were selected for further analysis. Domain search was performed for genes having lipase domain using CDD search [23].

### Subcellular localization prediction

Subcellular localization prediction for lipase genes was carried out using TargetP 1.1 [11] for all genes having lipase domain.

### Phylogenetic analysis

To further confirm the evolutionary relationships, a phylogenetic tree was constructed using sequences containing lipase domain from wheat and one reported lipase sequence from *Arabidopsis thaliana* and *Oryza sativa*. The complete genome sequences of *Oryza sativa* and Arabidopsis constitute a valuable resource for comparative genome analysis as they are representatives of the two major evolutionary lineages within the angiosperms. A number of articles had been reported in which genes of wheat have shown strong homology with Arabidopsis [29]. The phylogenetic tree was constructed using the neighbor-joining (NJ) method in MEGA v.10 [21] under the Jones-Taylor-Thornton amino acid matrix-based model of molecular evolution with uniform rates and pattern.

### Multiple sequence alignment

From the phylogenetic tree, sequences neighbor to lipase sequence of *Arabidopsis thaliana* [24] and *Oryza sativa* [38] were selected for alignment using Clustal Omega [35] and percent identity was also checked.

### Molecular modeling

It was found that protein sequences ensemble gene Id: TraesCS5B02G157100 and TraesCS3A02G463500 have the highest percent identity, and so these sequences were selected for molecular modeling in SWISS-MODEL [39], which is a homology-modeling server. By using the list of 50 templates, 3D models of lipase enzyme were constructed. Once the 3D models of lipase were built, the geometrical aspects of modeled protein structures were evaluated using Qualitative Model Energy Analysis (QMEAN) and models with the highest QMEAN value was selected for further work and the models are referred to as TraesCS5B02G157100 (Ensembl ID UPI0003D5866F) and TraesCS3A02G463500 (Ensembl ID Q8L6B0). Also, the Ramachandran plot for the models was generated for structure validation.

### Molecular dynamic simulation

The modeled structures were optimized using GROMACS 5.5 [1], which uses the Steepest Decent algorithm for minimization of the structure. Molecular dynamic (MD) simulation was carried out to understand the conformational behavior, structural details, and stability of protein complexes. Molecular dynamic (MD) simulation consists of an intensive force field calculation for each of the atom in a system, which is followed by an integration step, which advances the dynamical nature and positions of the atoms according to the classical laws of motion. MD simulation was used to unravel the stability analysis of protein complexes [33].

MD simulation of a modeled structure of lipase enzyme from wheat was performed using OPLS-AA force field [18], and all the protocols for the dynamic study were followed. In the TIP3P water model, the simulation was done and solvated in a cubic solvent box with a minimum distance of 1.0 nm. Till force tolerance of 1000 KJ mol^−1^ nm^−1^, the minimization of the structure was done. After energy minimization, the protein was equilibrated.

Equilibration is done in two phases. The first phase is NVT ensemble (constant number of particles, Volume, and Temperature), also referred to as “isothermal-isochoric” or “canonical” and it stabilizes the temperature of the system. The second phase is NPT ensemble, wherein the number of particles, pressure, and temperature are all constant, and this ensemble is also called the “isothermal-isobaric” ensemble—also it closely resembles the experimental conditions. Equilibration was done for 300 K temperature for 10 ns. Finally, molecular dynamics simulation was carried out for 50 ns at temperature 300 K. Different molecular dynamics parameters like the root mean square deviation (RMSD), root mean square fluctuation (RMSF), and radius of gyration (*R*_g_) were performed using the GROMACS tool. Origin 6.0 [10] was used for generating the plots.

#### Principal component analysis

To study the conformational change of proteins induced by inhibitor bindings, the principal component (PC) analysis has been widely used. The principal component analysis is also called the essential dynamics method or quasiharmonic analysis. It is one of the most popular methods as it systematically reduces the dimensionality of a complex system [40]. The principal component analysis (PCA) is mainly used to examine the relationship between different conformers or structures on the basis of their equivalent residues. The resulting principal component, orthogonal eigenvectors describe the axes of the maximal variance of the distribution of structures, and projection of distribution onto the subspace defined by the largest principal components results in a lower dimensional representation of the structural dataset. Also, the percentage of the total mean square displacement or variance of atom positional fluctuations captured in each dimension is represented by their corresponding eigenvalue [15]. In brief, the eigenvector represents the direction of motion of the protein and eigenvalues suggest amplitude of motion [7, 40].

PCA is performed on any high-dimensional dataset, so for the analysis of a protein trajectory, a C-matrix is constructed associated with a selected set of atomic positions. Most of the time, at the residue level, a coarse-grained description of protein motion is made by using the alpha carbon atom, which represents a point for the position of a residue. To get the eigenvectors and eigenvalues, it is mandatory to create the covariance matrix of the C-alpha atom’s fluctuation. The first and last eigenvectors were generated using PyMol tool and presented as a porcupine plot.

The equation which was used for the PCA plot generation is as follows:

$$ {P}_{xy}=\left\langle \left({m}_x(t)-{\left\langle {m}_x\right\rangle}_t\right)\left({m}_y(t)-{\left\langle {m}_y\right\rangle}_t\right)\right\rangle t $$

where, *m*_*x*_ and *m*_*y*_ represent the Cartesian coordinate of the *x*th atom and *y*th atom. “*t*” is the averaged time position of the complete trajectory [2, 21].

### Molecular docking

Molecular docking was performed using AutoDock v.4.2 [25]. The optimized structure of the lipase enzyme was retrieved after simulation in pdb format which was utilized for molecular docking. Tributyrin, a triglyceride, is an ester composed of butyric acid, and glycerol was used as a ligand for docking. Tributyrin (CID: 6050) was retrieved from the PubChem Database and was converted into the mol2 format using the Open Babel tool [27]. In AutoDock tool, the protein file as well as the ligand file was converted into PDBQT structure format for the docking process. All the necessary information required for AutoDock was stored in the PDBQT file. To the protein Kollman, charges were added and for protein as well as ligand Gasteiger, partial charges were kept constant during the process. During the whole docking process, phi, psi, and chi angles were treated as rotatable bonds. The docking grid box of dimension 60Å×60Å×60Å was made, covering the entire binding region for TraesCS5B02G157100 and similarly for TraesCS3A02G463500, 60Å×60Å×60Å grid box was made. The genetic algorithm was used for the conformational search strategy which was applied to the ligand as well as protein. The lamarckian genetic algorithm was used to study the free energy changes upon binding.

### Presentation and analysis software

UCSF Chimera v.1.13.1 [32] and PyMOL software [9] were used to visualize and analyze ligand–protein interactions. PyMOL was employed for better illustration of ligand–protein interactions for further analysis.

## Results and discussion

### Sequence retrieval and analysis

Data available at Ensembl Plants database was mined by using “lipase” as a keyword to search for all the lipase genes present in the annotated *Triticum aestivum* genome, and a list of 133 genes was obtained. Among the 133 genes initially retrieved, 62 genes code for lipoxygenase; therefore, these genes are not selected for further study. Lipoxygenases (LOXs; EC 1.13.11.12) are non-heme iron-containing dioxygenases widely distributed in plants and animals [34]. The remaining 71 genes were selected for further study.

### Motif and domain search

The amino acid motif, GXSXG, is commonly found in lipases. A lipase motif search analysis was performed for the proteins encoded by the remaining 71 lipase genes. The lipase motif was not encoded in 37 lipase genes, and these therefore not included for further study. The remaining 34 genes encoded proteins were found to consist of lipase motif in their deduced amino acid sequences. In CDD (Conserved Domain Database) search, it was found that out of 34 genes, 21 genes had a domain for lipase. CDD is a database having annotation for proteins, and it consists of multiple sequence alignment models for domains and full-length proteins.

### Subcellular localization prediction

Sub-cellular localization prediction was carried out using TargetP 1.1 for 21 genes (Table 1).

**Table 1:** Sub-cellular localization prediction of all the lipase genes

Ensemble ID	Len	cTP	mTP	SP	Others	Loc	RC
UPI0008442E28	405	0.191	0.114	0.281	0.133	S	5
UPI0008457161	405	0.110	0.052	0.584	0.097	S	3
UPI000DF5795D	420	0.177	0.122	0.276	0.122	S	5
UPI000842FC95	411	0.010	0.556	0.534	0.008	M	5
UPI000DF5B1A8	416	0.090	0.020	0.918	0.59	S	1
UPI0008448C8C	399	0.114	0.110	0.240	0.788	-	3
UPI000DF52C95	411	0.013	0.440	0.640	0.009	S	5
UPI000843C42A	340	0.007	0.084	0.986	0.005	S	1
A0A1D5UJB7	411	0.020.	0.318	0.489	0.040	S	5
UPI000DF53EBA	332	0.007	0.985	0.013	0.108	M	1
UPI0008437977	426	0.147	0.322	0.036	0.280	M	5
UPI000844AB89	411	0.273	0.464	0.011	0.245	M	5
UPI0008439BEA	398	0.007	0.084	0.986	0.005	S	1
UPI0003D5866F	414	0.006	0.075	0.987	0.006	S	1
Q8L6B0	350	0.002	0.364	0.805	0.039	S	3
UPI000DF57D77	446	0.083	0.020	0.913	0.065	S	1
UPI00098AEDB9	634	0.045	0.215	0.490	0.034	S	4
UPI000DF59195	575	0.038	0.155	0.663	0.031	S	3
UPI000581975B	634	0.038	0.155	0.663	0.031	C	3
UPI00098A6A95	815	0.022	0.092	0.165	0.018	S	5
UPI0008437DC2	815	0.019	0.090	0.192	0.029	S	5

NOTE: -*cTP* is the chloroplast transit peptide; mTP is a mitochondrial targeting peptide, *SP* is a signal peptide, present in the secretory pathway (S), chloroplast (C), or mitochondria (M); and RC stands for reliability class

On the basis of subcellular localization prediction, only one gene, i.e., UPI0008448C8C, is not reported in the secretory pathway. The reliability class ranges from 1 to 5, where 1 denotes the strongest prediction and vice versa. Reliability class is a measure of difference (“diff”) between the highest and the second highest output scores. The lower the value of RC, the safer is the prediction which can be observed from Table 1.

### Phylogenetic analysis

**Fig. 2:**
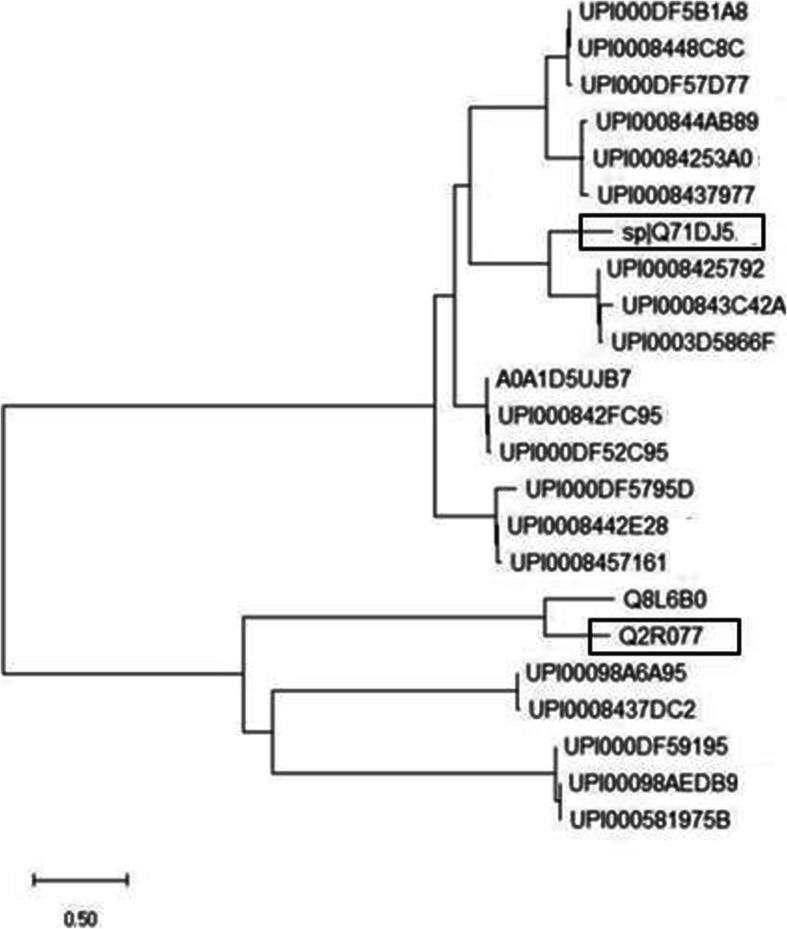
Phylogenetic tree of 21 lipase genes and one reported lipases gene from *Arabidopsis thaliana* and *Oryza sativa*

From the phylogenetic tree presented in Fig. 2, it can be predicted that wheat has a strong evolutionary relationship with *Arabidopsis thaliana* and *Oryza sativa*. Ensembl ID UPI0008425792 (uniport ID A0A1D5YD83), UPI000843C42A (uniport ID A0A1D5YD84), and UPI0003D5866F (uniport number W5FG08) were neighbors to the reported lipase sequence of *Arabidopsis thaliana*, and uniport number Q8L6B0 is neighbor to the reported lipase sequence of *Oryza sativa* [17].

### Multiple sequence alignment

MSA was done in Clustal Omega (refer to S1 and S2), and percent identity was also checked (Tables 2 and 3). Sequence UPI0003D5866F has 58.35% similarity with the reported lipase sequence of *Arabidopsis thaliana* (uniport number sp|Q71DJ5). Sequence Q8L6B0 has 51.74% with *Oryza sativa* gene (uniport number Q2R077). Ensembl ID UPI0003D5866F is coded by Gene ID TraesCS5B02G157100, and Ensembl ID Q8L6B0 is coded by TraesCS3A02G463500.

**Table 2:** Percent identity matrix (created by Clustal 2.1)

Ensemble ID	sp|Q71DJ	UPI000843C42A	UPI0003D5866F	UPI0008425792
sp|Q71DJ	100.00	52.38	58.35	58.10
UPI000843C42A	52.38	100.00	92.79	90.99
UPI0003D5866F	58.35	92.79	100.00	97.09
UPI0008425792	58.10	90.99	97.09	100.00

UPI0003D5866F is highly similar to *Arabidopsis thaliana* gene (58.35%)

**Table 3:** Percent identity matrix (created by Clustal 2.1)

Ensemble ID	Q8L6B0	Q2R077
Q8L6B0	100.00	51.74
Q2R077	51.74	100.00

Q8L6B0 is highly similar to *Oryza sativa* gene (51.74%)

### Molecular modeling

**Fig. 3:**
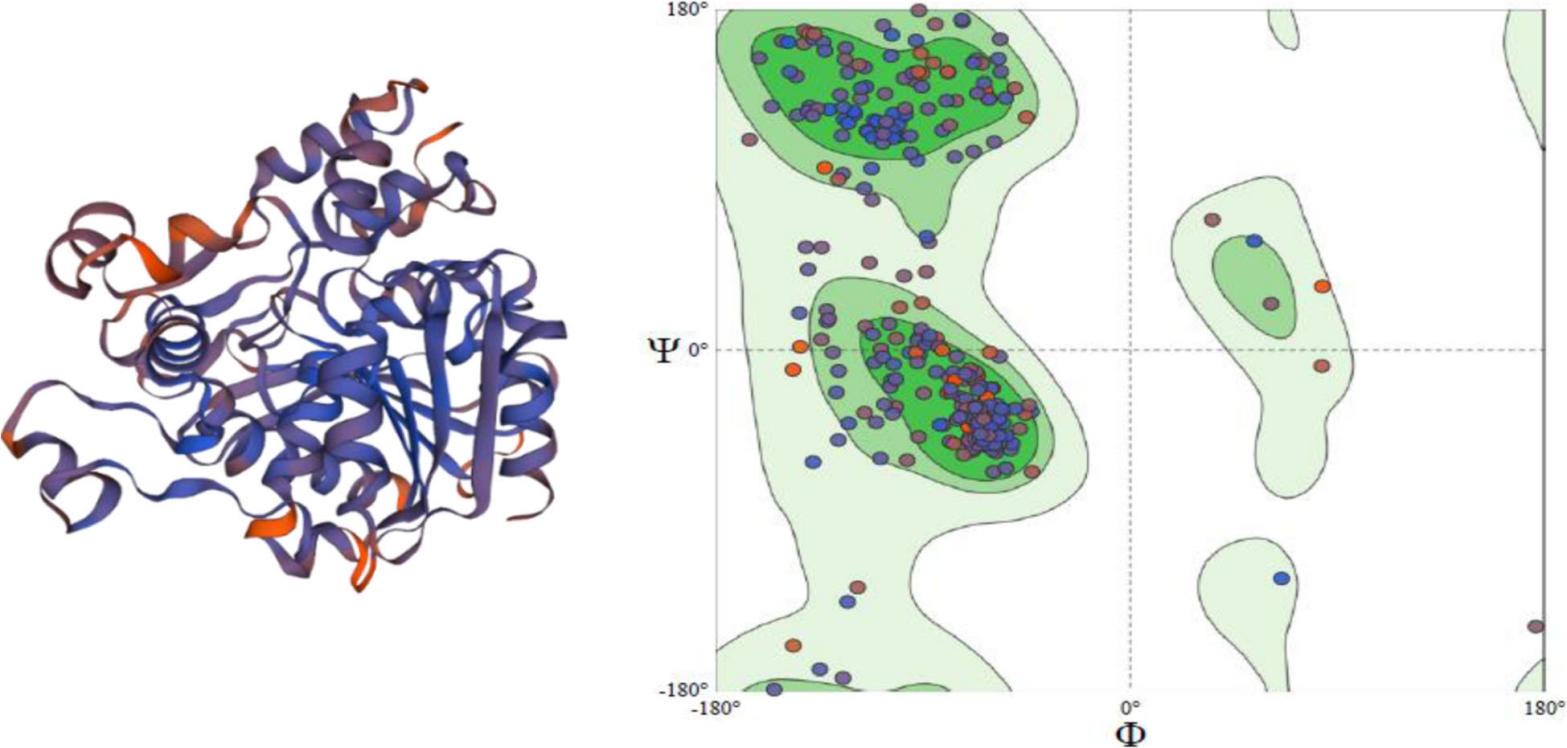
A 3D model of TraesCS5B02G157100, generated from SWISS-MODEL. Ramachandran plot of the model generated by SWISS-MODEL. 90.44% is in the favored region

For the ensemble gene Id TraesCS5B02G157100 and TraesCS3A02G463500, the molecular modeling was performed. The crystal structure of dog gastric lipase in complex with a phosphonate inhibitor (PDB id: 1k8q) having 30.14% sequence identity with TraesCS5B02G157100 was selected as a template to generate the 3D model (Fig. 3). QMEAN score was −4.37. Also, the modeled structure was validated by predicting the Ramachandran plot which indicated 90.44% is in the favored region (Fig. 3). MolProbity score is a combined protein quality score that gives the idea of crystallographic resolution at which such quality would be expected. In an ideal case, it should be as low as possible. MolProbity score for TraesCS5B02G157100 is 2.33 (Table 4) and for TraesCS3A02G463500 is 2.31 (Table 4) [24, 29].

**Table 4:** Table showing MolProbity results obtained after Ramachandran analysis

MolProbity Score	2.33	
Clash score	13.67	(A348 TYR-A375 TYR), (A338 ILE-A339 PRO), (A209 CYS-A384 PHE), (A135 TRP-A292 HIS), (A50 GLN-A54 PRO), (A225 ALA-A303 PHE), (A77 ILE-A106 PHE), (A345 TRP-A372 GLU)
Ramachandran Favored	90.44%	
Ramachandran outliers	0.55%	A318 LEU, A293 PRO
Rotamer outliers	1.59%	A282 ARG, A348 TYR, A226 VAL, A170 TYR, A52 LEU
C-beta deviations	4	A50 GLN, A288 GLU, A293 PRO, A292 HIS
Bad bonds	2/2977	A43 GLY, A292 HIS
Bad angles	57/4043	A288 GLU, A106 PHE, A215 ASP, A384 PHE, (A43 GLY-A44 GLY), A108 ASN, (A367 LEU-A368 ARG), A293 PRO, (A50 GLN-A51 LEU), (A370 THR-A371 PRO), A50 GLN, A356 ASP, (A318 LEU-A319 LEU), A368 ARG, (A53 LEU-A54 PRO), A292 HIS, (A38 VAL-A39 PRO), A344 MET, A68 ASP, A219 ALA, A253 VAL, A76 HIS, A129 ASN, A209 CYS, A339 PRO, (A175 SER-A176 LYS), A311 PHE, A229 HIS, A275 ASN, A225 ALA, (A221 PHE-A222 VAL), A62 HIS, A96 HIS, A177 ILE, A335 LEU, A222 VAL, A182 HIS, A334 ASP, A261 HIS, A285 HIS, A193 PHE, A145 HIS, A91 PRO, A139 HIS
Cis prolines	1/16	(A290 GLU-A291 PRO)

**Fig. 4:**
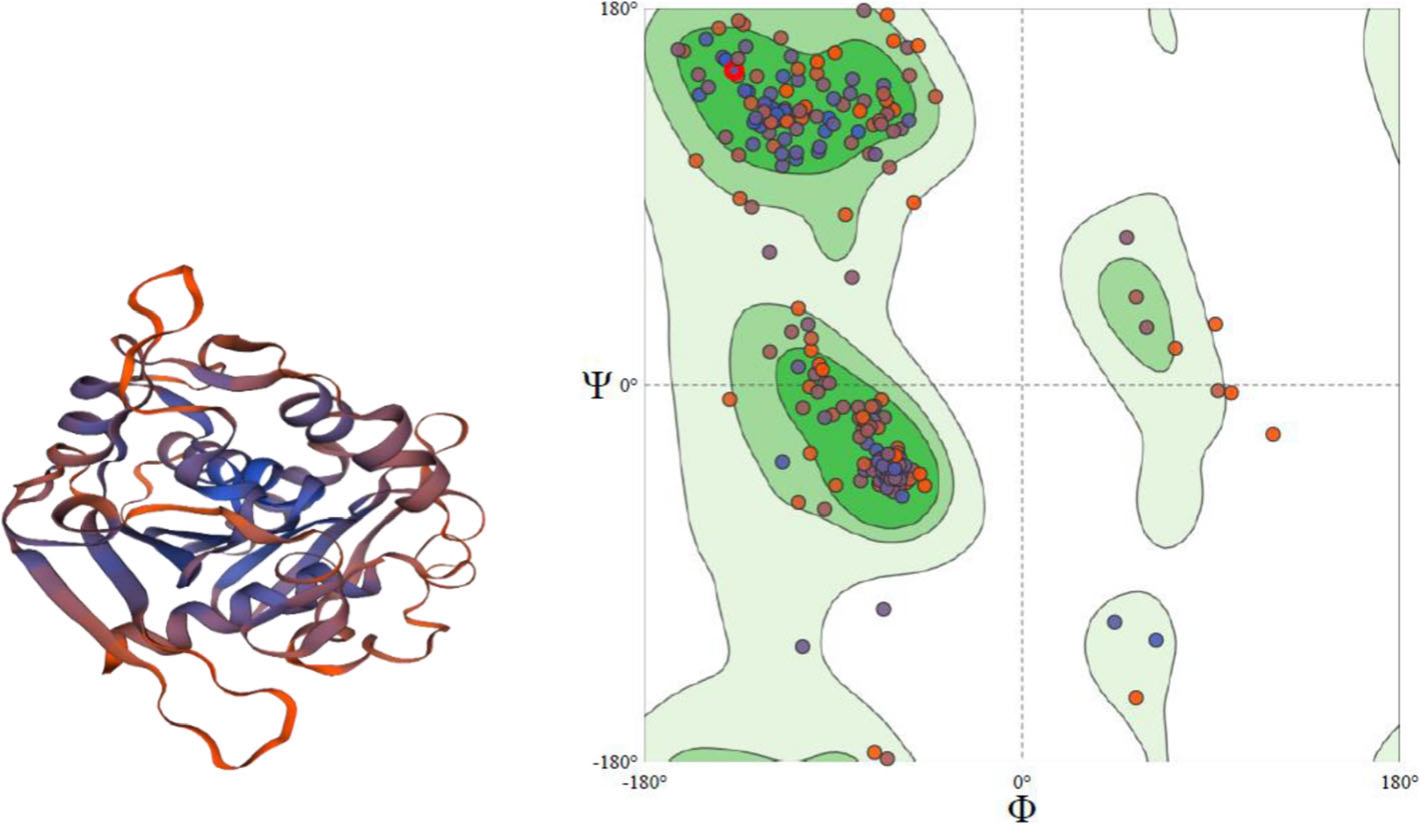
A 3D model of TraesCS3A02G463500, generated from SWISS-MODEL. Ramachandran plot of model generated by SWISS-MODEL. 89.89% is in the favored region

Similarly, the crystal structure of *Rhizomucor miehei* triacylglyceride lipase (PDB id: 3TGL) of 30.95% sequence identity with wheat gene was selected as the template for the TraesCS3A02G463500 sequence extracted from Ensembl database to generate the 3D model (Fig. 4). QMEAN score was −2.18. Also, the modeled structure was validated by predicting Ramachandran plot which indicates 89.89% in favored region (Fig. 4) followed by a detailed analysis listed in Table 5. Thus, the predicted modeled structure can be considered of good quality, and it was used for molecular docking studies with triglycerides as a ligand.

**Table 5:** Table showing MolProbity results obtained after Ramachandran analysis

MolProbity score	2.31	
Clash score	11.59	(A216 PHE-A251 PHE), (A174 HIS-A302 MET), (A183 PHE-A207 VAL)
Ramachandran favored	89.89%	
Ramachandran outliers	3.0%	A131 MET, A68 GLY, A245 GLN, A60 PHE, A148 THR, A147 THR, A220 LEU, A251 PHE
Rotamer outliers	1.74%	A119 LEU, A189 VAL, A45 VAL, A162 LYS
C-beta deviations	5	A60 PHE, A251 PHE, A142 SER, A148 THR, A233 VAL
Bad bonds	2/2175	A251 PHE-A252 PRO, A250 HIS-A251 PHE
Bad angles	33/2966	(A220 LEU-A221 PRO), A90 TYR, A251 PHE, (A131 MET-A132 PRO), (A57 THR-A58 GLN), A69 ASP, A149 ILE, A235 HIS, A79 ILE, (A233 VAL-A234 PRO), A142 SER, A240 PHE, (A243 PHE-A244 PRO), A303 HIS, A189 VAL, A222 HIS, A174 HIS, A89 ALA, A85 ASN, A80 ILE, (A204 GLN-A205 PRO), A81 VAL, A258 HIS, A239 TYR, A249 HIS, (A251 PHEA252 PRO), (A288 GLY-A289 ASN), A250 HIS
Cis non-proline	2/257	(A57 THR-A58 GLN), (A288 GLY-A289 ASN)
Cis prolines	1/11	(A236 LEU-A237 PRO)
Twisted prolines	1/11	(A251 PHE-A252 PRO)

### Molecular dynamic simulation

The stability and analysis of the native protein structure were performed by surrounding them into a cubical box at a temperature of 300 K that was maintained computationally. Various computational analyses were carried out to evaluate the stability of the system. The stability of lipase enzyme from wheat’s gene was evaluated with several time-dependent structural parameters (like RMSD, RMSF, R_g_) which was obtained from the 50-ns molecular dynamics simulation [22].

#### RMSD

**Fig. 5:**
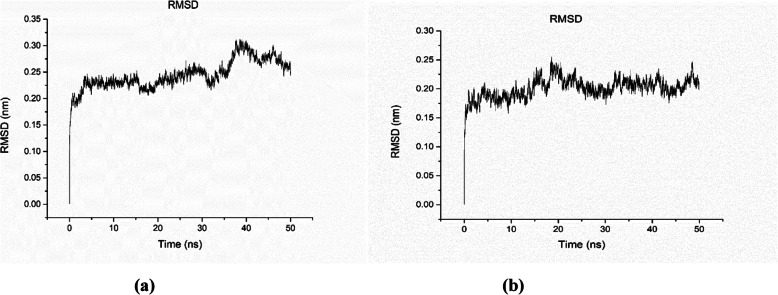
The RMSD vs. time plot for lipase enzyme model coded by TraesCS5B02G157100 **a** and TraesCS3A02G463500 **b**, respectively

The root mean square deviation (RMSD) is used to measure the difference between the backbones of a protein structure, from its initial structural conformation to its final position. From the deviations produced during the course of its simulation, the stability of the protein structure, relative to its conformation can be determined. Smaller deviations indicate a more stable protein structure [33]. Figure 5a, b shows the plot of RMSD vs. time (ns) for TraesCS5B02G157100 and TraesCS3A02G463500, respectively. Fluctuation can be observed with an average value of 0.25 nm, which is due to the flexibility of several loops and α-helix. The RMSD value fluctuates between 0.15 nm and 0.32 nm with an average of 0.23 nm.

#### RMSF

**Fig. 6:**
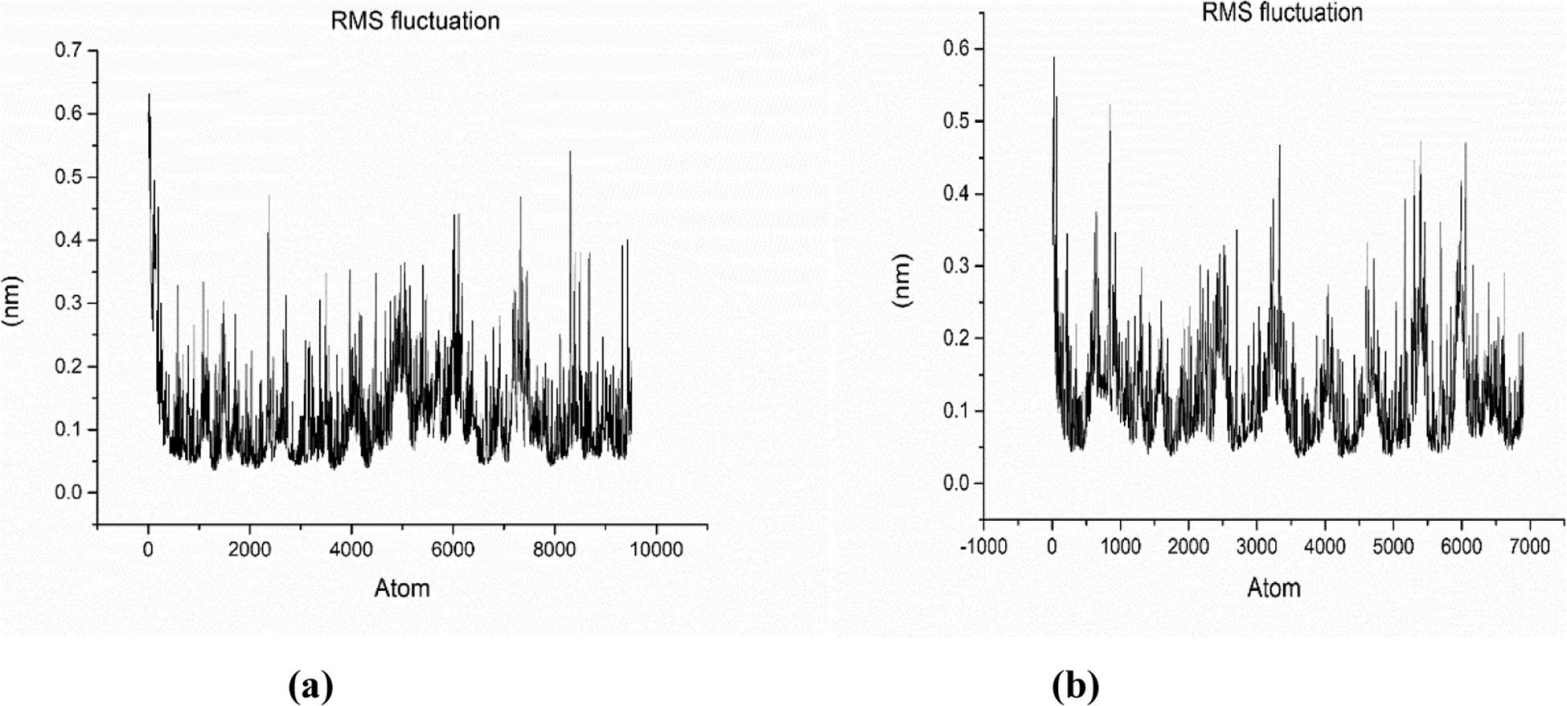
The RMSF vs. time plot for lipase enzyme model coded by TraesCS5B02G157100 **a** and TraesCS3A02G463500 **b**, respectively

**Fig. 7:**
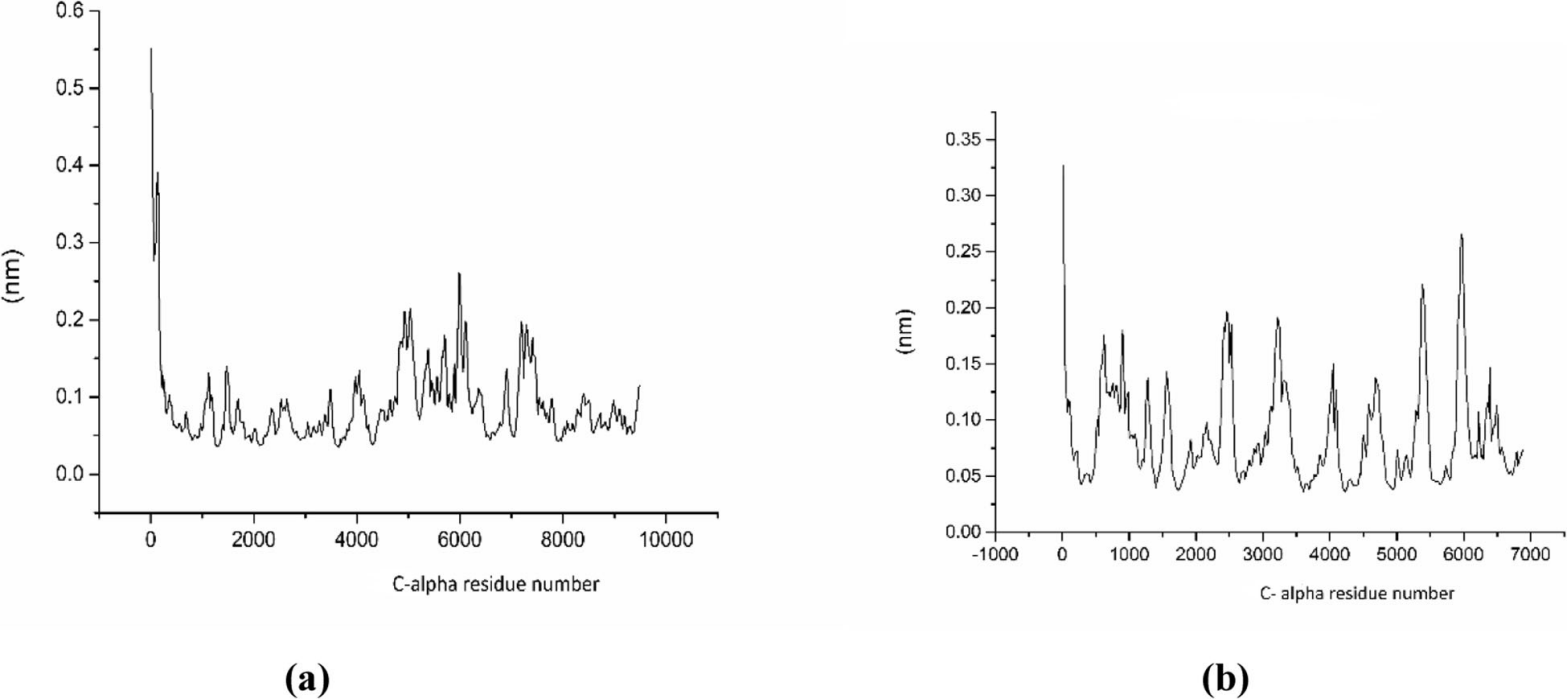
The Cα-RMSF vs. time plot for lipase enzyme model coded by TraesCS5B02G157100 **a** and TraesCS3A02G463500 **b**, respectively

The root mean square fluctuation (RMSF) plots provide us information about the flexible regions of the protein complexes. In proteins, helical and sheet structures show lower RMS fluctuation as compared to the loop, turns, and coils. The lower RMSF value indicates the well-structured regions, whereas the higher RMSF value indicates loosely organized loop or terminal ends. The RMSF plot for lipase enzyme model coded by TraesCS5B02G157100 (Fig. 6a) and TraesCS3A02G463500 (Fig. 6b) is presented. All of the RMSF values were small and below 1nm, indicating stability of the protein [26]. The RMSF value for the Cα backbone was also calculated for 50 ns simulation in order to evaluate the stability of the structure mainly Cα atoms. The RMSF value for the Cα residue plot for TraesCS5B02G157100 and TraesCS3A02G463500 is presented in Fig. 7a ,b, respectively.

#### Radius of gyration (*R*_g_)

**Fig. 8:**
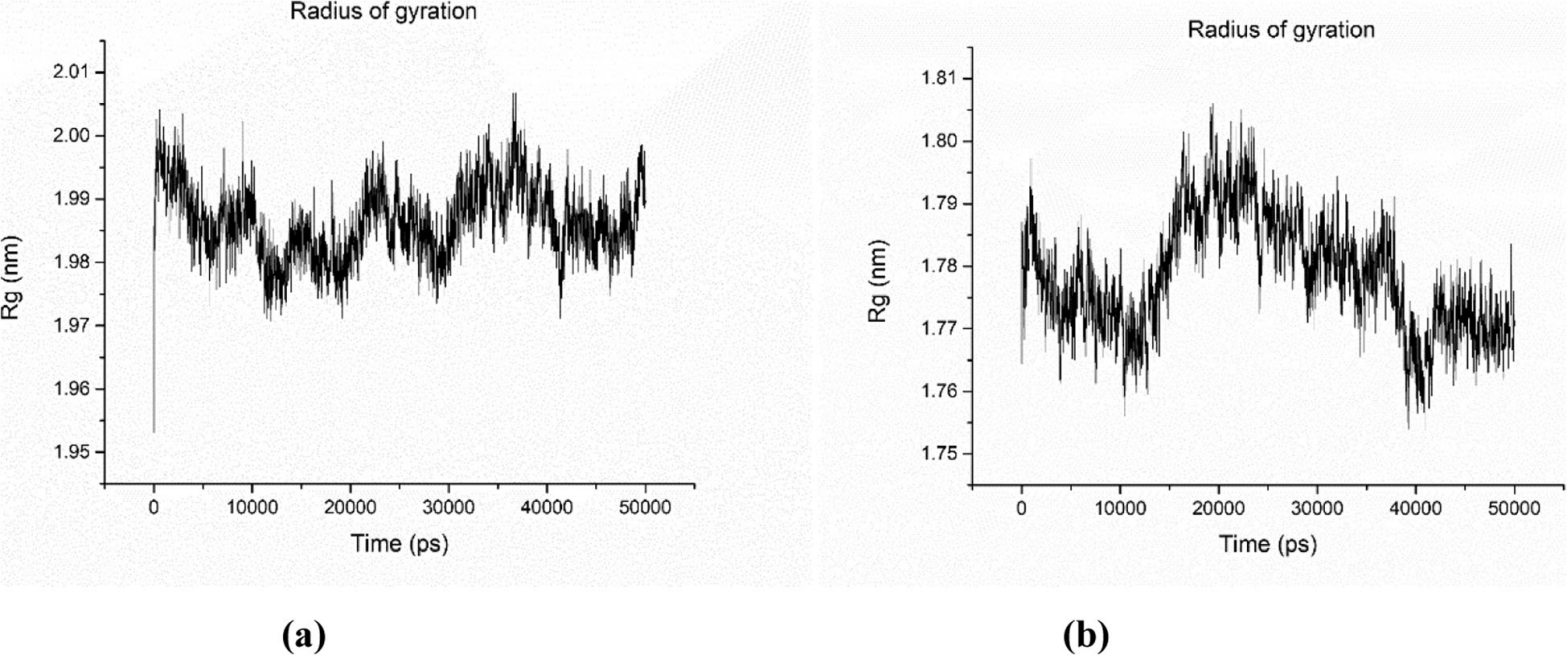
The radius of gyration (*R*_*g*_) vs. time plot for lipase enzyme model coded by TraesCS5B02G157100 **a** and TraesCS3A02G463500 **b**, respectively

The radius of gyration (*R*_g_) was determined to understand the level of compaction in the structure of the enzyme. The *R*_g_ value is assigned as the mass-weighted RMSD fit of a collection of atoms from their common center of mass [33]. Figure 8 describes the compactness of the structure during a complete simulation. The *R*_g_ plot for the lipase enzyme model coded by TraesCS5B02G157100 and TraesCS3A02G463500 is shown in Fig. 8a, 8b, respectively.

#### Principal component analysis

**Fig. 9:**
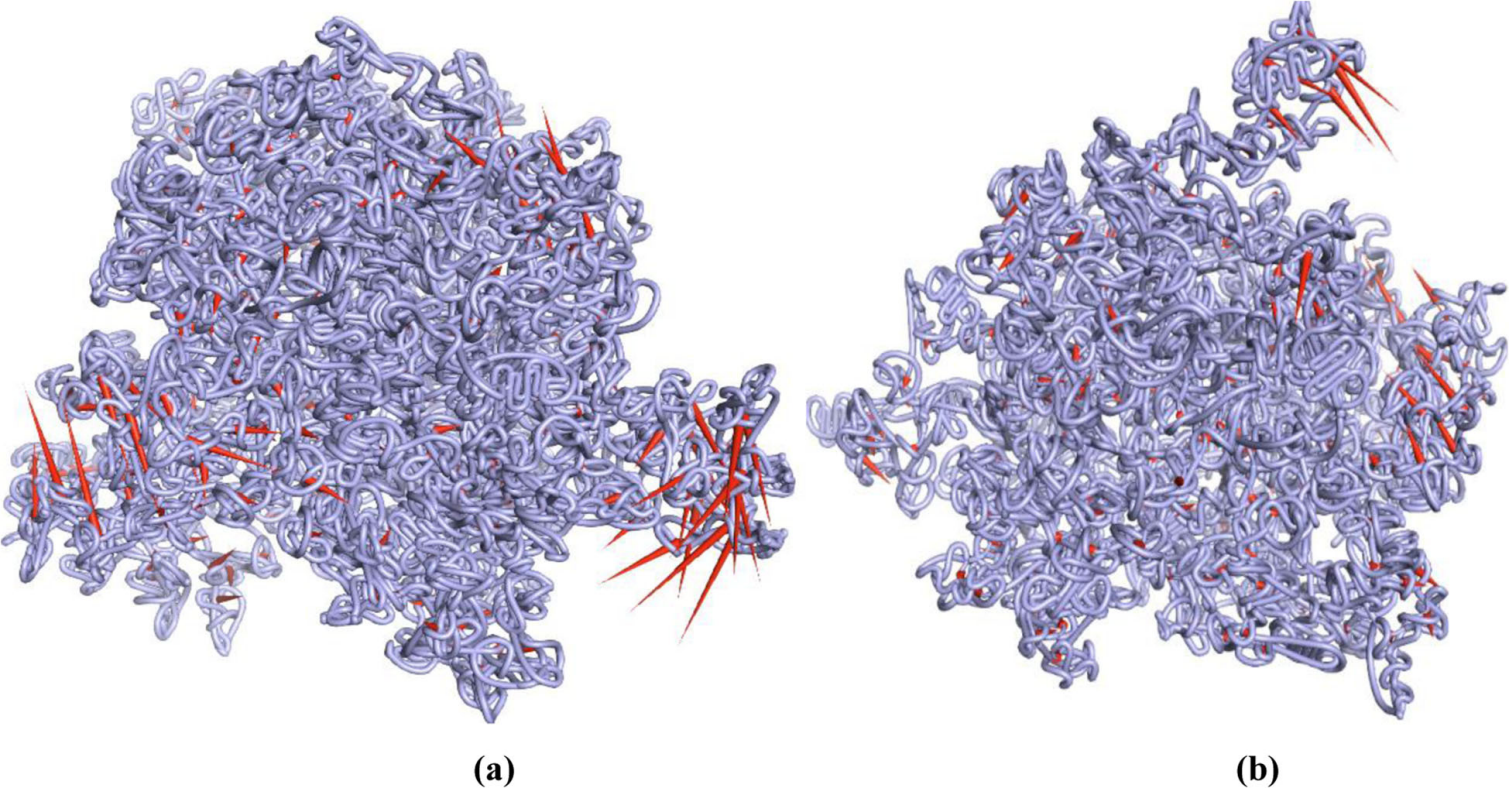
Principal component analysis (PCA). The graph showing porcupine plot depicting the movement of Cα atoms in crystal structures of lipase enzyme model coded by TraesCS5B02G157100 **a** and TraesCS3A02G463500 **b**

In this current study, PC analysis was carried out to get the detailed insight into the concerted motions of lipase enzyme based on the equilibrium phase of MD simulations. In order to qualitatively understand the differences in a motional pattern, a porcupine plot was generated by performing the extreme projections of MD trajectories. To visualize the movement of the backbone, the “Mode vectors” present in the PyMol software were used and a porcupine plot was generated (Fig. 9) by aligning the trajectories over the original protein structure. The direction of the arrow (red color) is indicative of the direction of motions, and the length of the arrow reflects the strength of the movements.

### Molecular docking

**Fig. 10:**
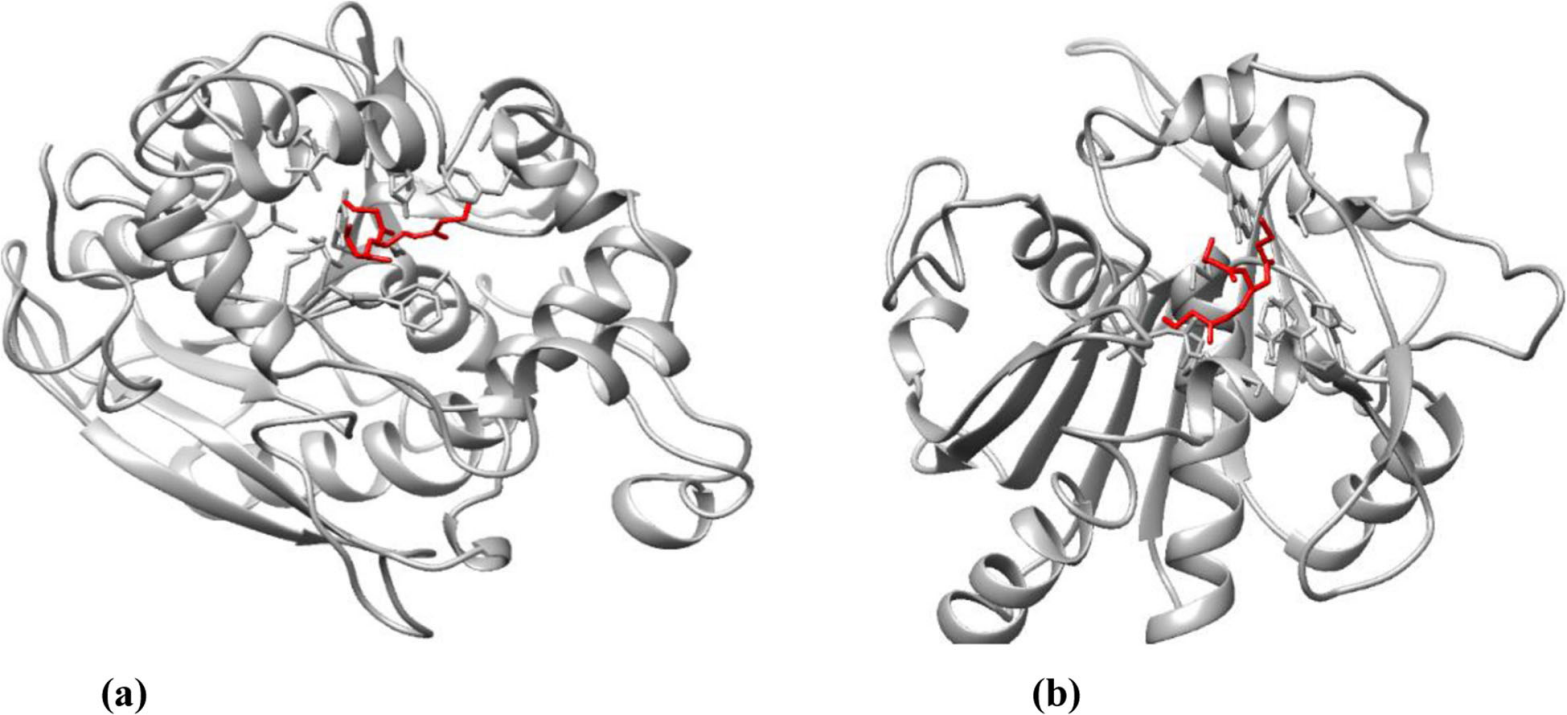
Molecular interaction analysis of tributyrin with lipase enzyme coded by TraesCS5B02G157100 **a** and TraesCS3A02G463500 **b**, visualized in UCSF Chimera. The ligand tributyrin is shown in red color, whereas the model of lipase enzyme is shown in gray color

The optimized modeled structure obtained from MD simulation was further used for molecular docking with tributyrin (triglyceride, CID: 6050). Docking results predicted extensive interactions between ligand and catalytic site. Protein–ligand complex structures with the lowest docking binding energy were selected. The binding energy for tributyrin with a modeled structure of a lipase enzyme coded by wheat TraesCS5B02G157100 (catalytic site: 181–185, GHSQG) and TraesCS3A02G463500 (catalytic site: 173–177, GHSMG) were −9.83 kcal/mol and −6.67 kcal/mol, respectively (Table 6). The interaction of the ligand with the enzyme was observed in UCSF Chimera v.1.13.1 which is shown in Fig. 10.

**Table 6:** Binding affinity of protein with tributyrin

Protein	H-bond acceptor	H-bond donor	Binding affinity (kcal/mol)	H-bond distance (Å)
TraesCS5B02G157100	LIGI:O	SER183: HG	−9.83	2.2
TraesCS3A02G463500	LIGI:O	HIS174: HE2	−6.67	2.6

**Fig. 11:**
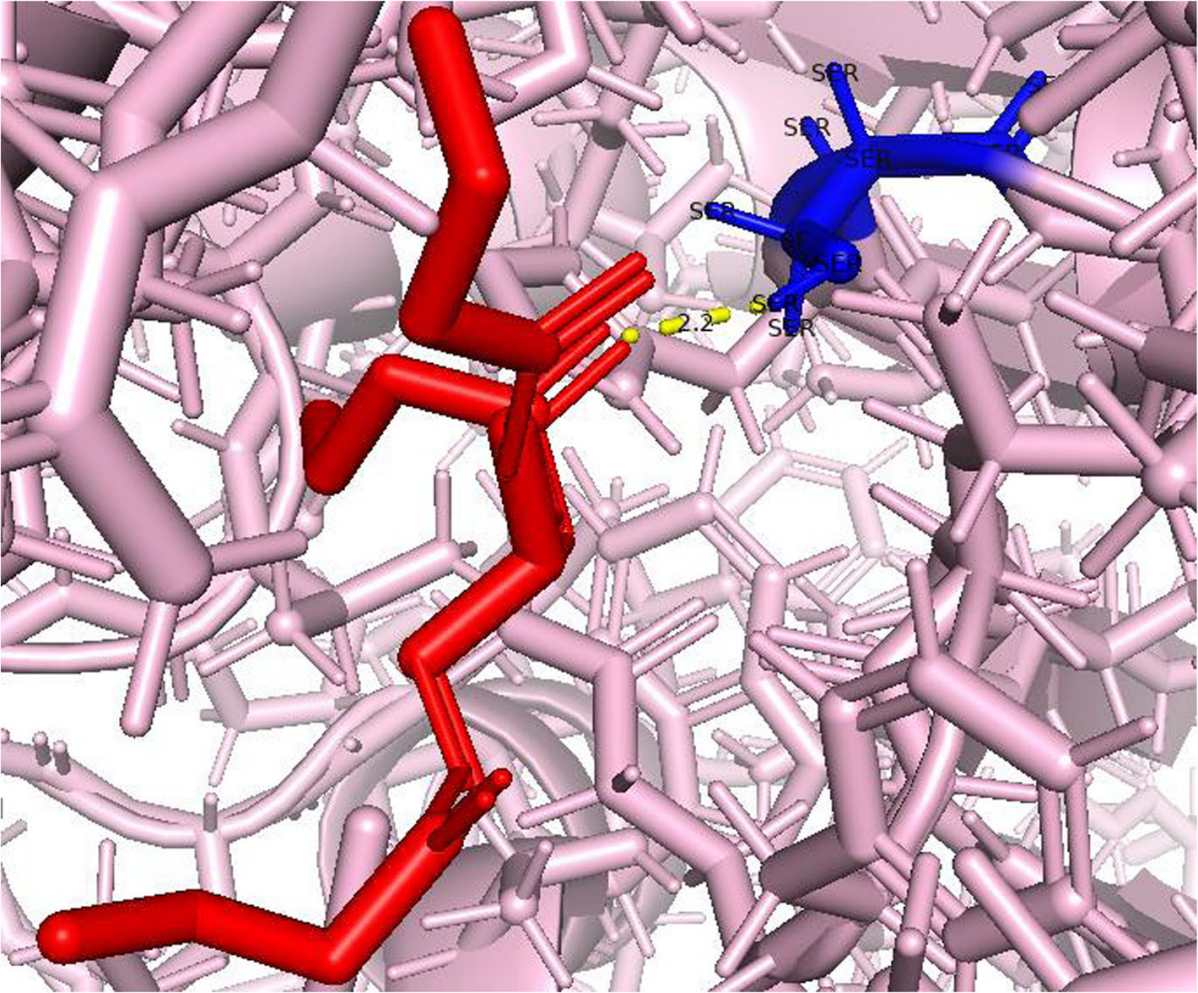
Hydrogen bond formation by Tributyrin (shown in red color) attached with a catalytic site (181–185, GHSQG) at SER183

**Fig. 12:**
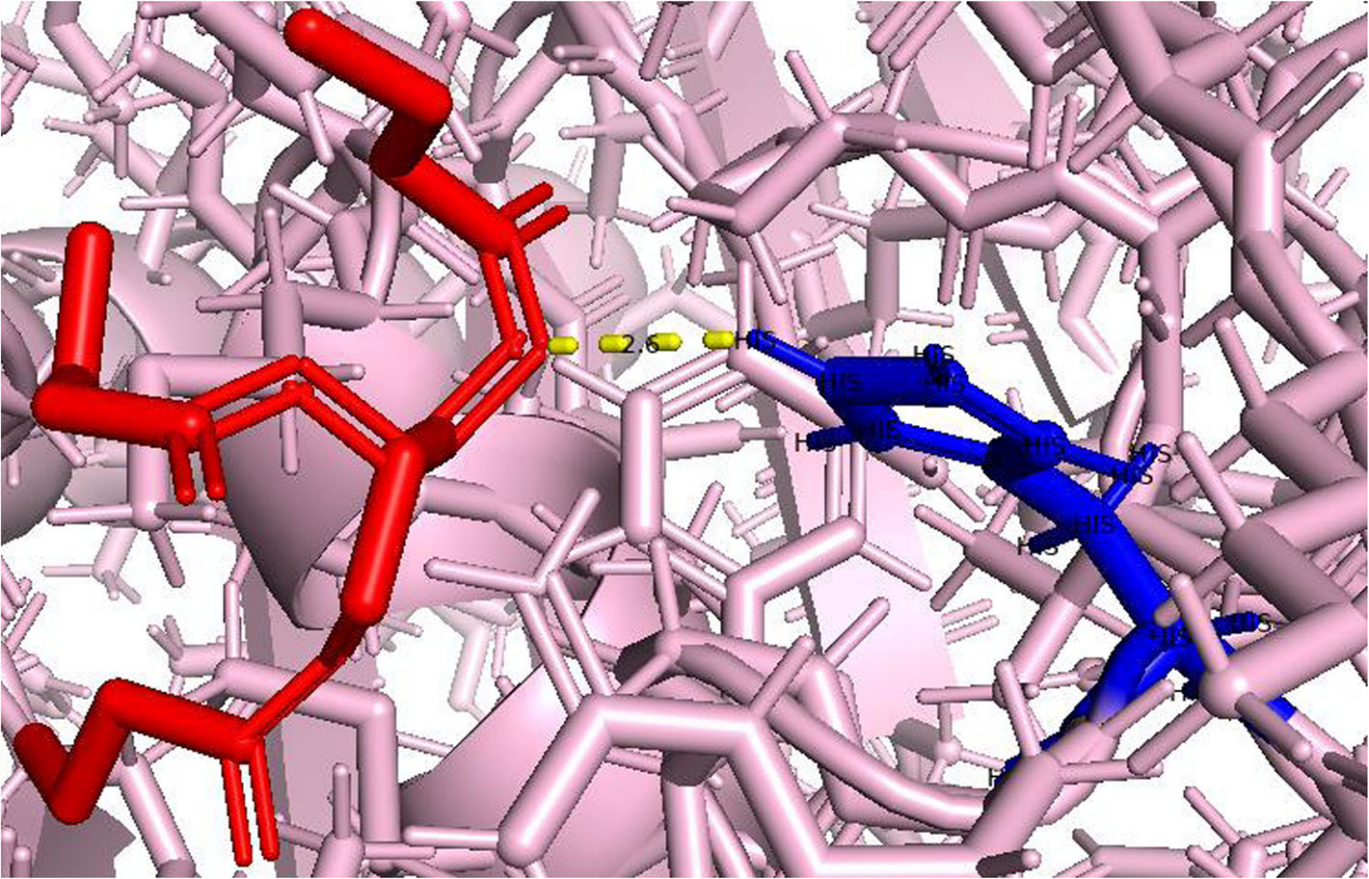
Hydrogen bond formation by Tributyrin (shown in red color) attached with a catalytic site (173–177, GHSMG) at HIS174

The H-bond distance between the ligand and catalytic triad was explored using the Pymol visualization tool. The modeled structure of lipase enzyme coded by TraesCS5B02G157100 is attached with the ligand by hydrogen bond of distance 2.2 Å (Fig. 11), and lipase enzyme coded by TraesCS3A02G463500 has hydrogen bond distance of 2.6 Å with the ligand (Fig. 12).

## Conclusion

Data available at Ensembl Plants database was mined to search for all the lipase genes from annotated *Triticum aestivum* genome and a list of lipase genes was obtained. Among the list of genes retrieved excluding genes code for lipoxygenase, a motif finding and CDD search were performed to search for the GXSXG motif. Sub-cellular localization prediction was carried out, and a total of 21 lipase genes were finally screened down which was found to be present in a secretory pathway. Further evolutionary relationship predicted ensemble genes ID TraesCS5B02G157100 and TraesCS3A02G463500 may have a strong evolutionary relationship with *Arabidopsis thaliana* and *Oryza sativa.* Thus, these sequences were modeled and docked with tributyrin, and binding efficiency of −9.83 kcal/mol and −6.67 kcal/mol, respectively, was observed. Several analysis methods were employed for trajectory analysis, including RMSD, RMSF, *R*_g_ calculation, interaction energy calculation, and PC analysis. Both the protein sequences are expected as a signal peptide, i.e., they are involved in the secretory pathway. So they can be easily purified and can be further used for research work. This work provides us a basic understanding of the gene encoding lipase in the wheat genome.

## Data Availability

The data and materials are original and available.

## Abbreviations

EC: Enzyme commission
QMEAN: Qualitative Model Energy Analysis
RMSD: Root mean square deviation
RMSF: Root mean square fluctuation
MD: Molecular dynamic
CDD: Conserved Domain Database
